# Is dystrophin immunogenicity a barrier to advancing gene therapy for Duchenne muscular dystrophy?

**DOI:** 10.1038/s41434-025-00531-y

**Published:** 2025-04-03

**Authors:** Dariusz C. Górecki, Pawel Kalinski, Joanna Pomeroy

**Affiliations:** 1 School of Medicine, Pharmacy and Biomedical Sciences, University of Portsmouth, St Michael Bld, White Swan Road, Portsmouth, PO1 2DT UK; 2 Department of Immunology, Roswell Park Comprehensive Cancer Center, Elm and Carlton Streets, Buffalo, NY 14263 USA

**Keywords:** Gene therapy, Inflammation

## Abstract

Duchenne muscular dystrophy (DMD) is a neuromuscular disorder that leads to severe disability and premature death in young men. As DMD is caused by the absence of dystrophin, therapeutic development has focused on strategies to restore dystrophin expression. These include readthrough of premature stop codons, exon skipping to restore the reading frame, and gene therapy. The first two methods are mutation-specific, benefiting only subsets of patients, whereas gene therapy could treat all individuals with DMD. Immunogenicity of dystrophin may challenge these efforts. The immune system can recognize dystrophin as a neo-antigen, just as it can recognize newly arising antigens present on mutated cells. An in-depth evaluation of anti-dystrophin immune response as a factor affecting the treatment effectiveness is needed. Key questions include the underlying mechanisms of immunity induction by antigenic epitopes of the re-expressed dystrophin, the impact of such responses on the therapeutic efficacy, and the role of patient-specific risk factors, such as preimmunization due to revertant fibres, chronic muscle inflammation, pre-existing T lymphocytes reactive to dystrophin, which avoided deletion in dystrophic thymus, or antigen cross-reactivity. Patients’ immune status assessment before treatment may help mitigating anti-dystrophin responses. Exploring potential therapeutic strategies to enhance treatment outcomes is also essential: Since DMD can be diagnosed at birth, early dystrophin re-expression could prevent damage and also potentially induce neonatal tolerance. In older patients, carefully managed immunosuppression and tolerogenic protocols could pave the way for more successful dystrophin replacement therapies.

## Introduction

On June 20, 2024, the U.S. Food and Drug Administration expanded its approval of Elevidys (delandistrogene moxeparvovec), a gene therapy for Duchenne muscular dystrophy (DMD), to include both ambulatory and non-ambulatory individuals aged 4 years and older with a confirmed DMD gene mutation. This decision has been hailed as a milestone in the quest to cure this debilitating and currently incurable disease. Unfortunately, the recently published results from the Phase 3 EMBARK study revealed that treatment with delandistrogene moxeparvovec did not result in significant improvement in the primary endpoint, nor were there statistically significant differences observed in the secondary endpoints [1].

DMD is an X-linked genetic disease [2]. It presents already at the embryonic stage [3, 4] with functional abnormalities present in pre-symptomatic DMD infants [5], and the affected boys typically diagnosed at 2–3 years of age. Progressive muscle degeneration and wasting lead to loss of ambulation, usually at 10–12 years, and the need for assisted ventilation in 20s. Despite recent progress in symptom management, most DMD men die between 20 and 40 years of age due to cardiac and/or respiratory failure [6]. Pathology involves progressive degeneration of skeletal and heart muscles aggravated by sterile inflammation [7]. The pleiotropic effects of the mutant gene also include neuropsychiatric deficits [8–10] and bone mineral abnormalities, the latter further exacerbated by chronic steroid therapy [11–13].

These widespread symptoms stem from the complexity of this large human gene, which contains at least eight promoters that drive the expression of its tissue-specific and structurally distinct isoforms. Mutations affecting full-length (427 kDa) dystrophins are necessary and sufficient to trigger the disease, whereas the loss of additional, truncated isoforms (Dp260, Dp140, Dp116 and Dp71) can exacerbate the pathology, often in a tissue-specific manner. The dystrophin-null muscle phenotype is rare and poorly characterised, but appears to be aggravated in both humans [14] and a mouse model [15].

Mutations in the DMD gene can also cause Becker muscular dystrophy (BMD), which has a significantly later onset and slower progression than DMD does. These different outcomes are due to variations in the underlying molecular mechanisms. The rare BMD mutations preserve the transcript reading frame, resulting in internally truncated but semi-functional proteins (mini- or micro-dystrophin) that retain both the N- and C-termini. Their presence results in a milder disease [16] and such mini- or micro-dystrophins are exploited in current therapeutic approaches (as discussed below).

DMD mutations disrupt the reading frame, leading to the absence of full-length dystrophin. In healthy muscles, this dystrophin is found primarily under the sarcolemma (cell membrane) of myofibers. Its N-terminus binds to cytoskeletal F-actin, and the central rod domain, which is composed of spectrin-like repeats and proline-rich hinges, provides elasticity, whereas the C-terminal regions interact with dystrophin-associated proteins (DAPs), which play roles in binding extracellular matrix molecules and scaffolding for several signalling proteins [17].

However, the role of Dp427 extends beyond serving as a scaffold linking the myofiber cytoskeleton, the cell membrane and the ECM. In fact, the expression of DMD gene transcripts is widespread and shares characteristics with that of housekeeping genes [18]. The loss or dysregulation of its expression also leads to a range of cell-specific abnormalities in non-muscle tissues. These include the brain, bone, epithelia, and even malignant tissues, where alterations in DMD expression can serve as a prognostic marker for survival [18, 19].

Additionally, in muscle, full-length dystrophin is essential throughout the entire muscle life cycle, both in developing and postnatal muscle: Dp427 is important in muscle stem (satellite) cells [20] and in myoblasts [21] where it regulates the asymmetrical division of muscle stem cells, myoblast proliferation, migration, and finally differentiation into fully functional, stable myofibres [22, 23]. In mature muscle fibres, dystrophin prevents contraction-induced injury [24], but its loss in fully differentiated myofibres does not seem to cause dystrophic damage [23, 24]. This multicellular role is both an additional challenge and also an opportunity when therapeutic approaches are considered. Targeting dystrophin to myogenic cells could evoke lasting expression, and gene targeting to dividing myoblasts is more effective than targeting to postmitotic myofibers. Finally, the use of CRISPR/Cas9 technologies for DMD gene repair in muscle stem cells could lead to the generation of healthy satellite cells and the repopulation of muscles with functional myofibres [25].

## Dystrophin re-expression: Targeting the root cause of DMD

The view that DMD pathology is caused by the absence of dystrophin in myofibres, making them unable to withstand normal contraction forces, has driven treatment efforts towards restoring dystrophin expression there [26]. The rationale has been that re-expression of dystrophin would address the primary cause of this disease. This approach is being pursued through several strategies. One method involves the use of drugs such as Ataluren, which promote ribosomal readthrough of premature stop codons in a subset of patients with specific mutations that respond to this treatment [27].

The second approach leverages the observation that BMD patients with mutations preserving the transcript reading frame, thus resulting in truncated mini- and micro-dystrophins, exhibit significantly milder phenotypes. Drugs that induce exon skipping are intended to convert out-of-frame mutations in DMD into in-frame mutations, thereby promoting widespread re-expression of internally truncated but semi-functional mini- and micro-dystrophins [28]. This approach also applies to subsets of patients with mutations amenable to reading-frame restoration. Notably, exon skipping drugs are not approved in the EU due to insufficient evidence of statistically significant clinical benefits.

Recently, gene therapy involving the use of viral vectors to re-express dystrophin has dominated the scene [29]. Theoretically, this approach is suitable for all DMD patients, regardless of their mutation type. Notably, owing to the current loading capacity of viral vectors and the large size (14 kb) of the full-length dystrophin cDNA, this method also employs mini- and micro-dystrophins. Consequently, this treatment converts DMD into a milder BMD-like condition. Nevertheless, expression of mini-dystrophin has a potential to improve muscle functions, as seen in a transgenic mouse model [30] and in patients [29], and with improved technologies, re-expression of full-length dystrophin should be achievable in the future [31].

## Dystrophin as an antigen

However, the primary challenge with this approach lies in the immunogenicity of both the viral vectors and the re-expressed dystrophin protein. The vector immunogenicity is a well-recognised problem that has been extensively studied and is likely mitigated through the development of improved, less immunogenic vectors. Therefore, this aspect is not discussed here. However, the more pressing and complex issue of the immunogenicity of the re-expressed dystrophin itself remains. This challenge persists even with the use of low-immunogenicity vectors [32] or with vector-less exon skipping [33] as the immune system may still recognise dystrophin as foreign, leading to an immune response that could undermine therapeutic effectiveness [34, 35].

Unfortunately, this aspect has not been given sufficient attention until now. Exon skipping and gene therapy approaches were somehow assumed not to trigger significant immune responses against the re-expressed dystrophin, and often this possibility was not evaluated at all. Yet, it may occur, as manifested clinically before [36], and as the severe adverse events in recent DMD clinical trials [37].

Addressing dystrophin immunogenicity is therefore critical to the success of gene therapies. The key questions are **why and to what extent the re-expressed dystrophin triggers immune responses and how badly these responses can suppress the therapeutic effect and damage muscles**. This phenomenon needs to be considered in relation to gene therapy, and with the utmost caution when pursuing CRISPR/Cas9 technology to repair the DMD gene in satellite cells of adult patients because the immune response to dystrophin could eliminate muscle stem cells and disrupt muscle regeneration. Crucially, recognising this problem should not impair but rather improve prospects for all kinds of dystrophin-replacement therapies.

The immune system distinguishes between self and nonself, which is essential for effective immunity to foreign antigens while avoiding harmful autoimmune responses to the “self”.

This can be illustrated by comparing gene therapies for DMD vs. spinal muscular atrophy (SMA). The main difference lies in the presence of two survival motoneuron (SMN) genes, where SMN2 expresses mostly unstable but also small amounts of functional SMN protein. The presence of the traces of protein instructs the developing immune system to recognize it as “self”. Therefore, the SMN generated through gene therapy does not trigger immune responses.

**Fig. 1:**
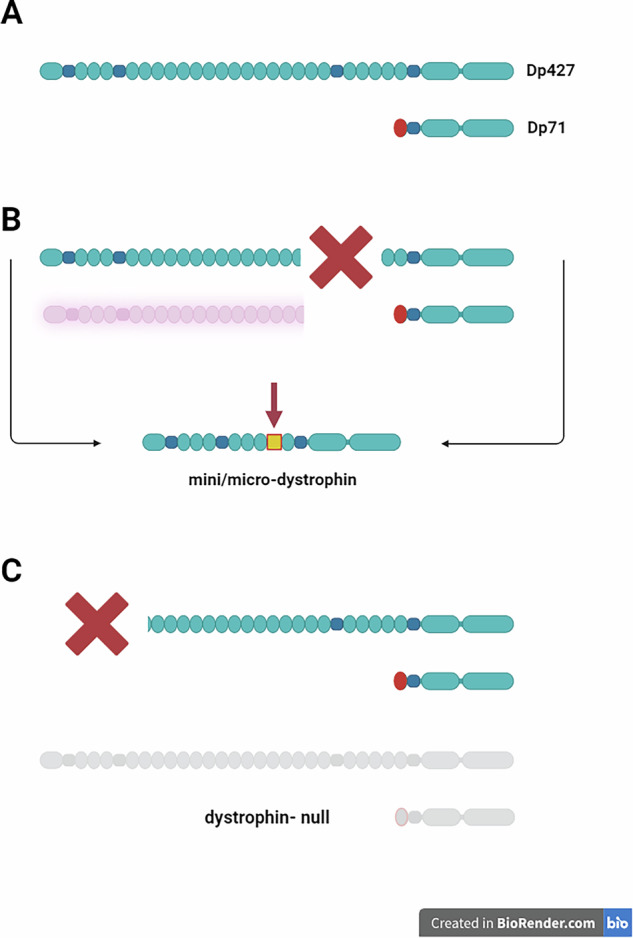
Dystrophin proteins and their antigenic epitopes in DMD. **A** Healthy individuals express full-length dystrophins (Dp427) and progressively truncated isoforms, of which Dp71 is shown here. All dystrophins share a common C-terminus. **B** The majority of DMD patients have mutations disrupting protein translation in the middle of the protein, while the expression of Dp71 is unaffected. In these cases, translation of short, unstable N-terminal dystrophin polypeptides might be possible (shown in pink), and these transient peptides could train the immune system to recognise corresponding dystrophin regions as self. The revertant fibres express mini/micro-dystrophin containing the N- and C-terminal domains connected by a junctional region (yellow square) not present in any endogenous dystrophin, and therefore potentially antigenic. **C** Patients with exon 3--9 mutations cannot express any full-length dystrophin or N-terminal peptides, but they do express short isoforms. In contrast, dystrophin-null patients lack any dystrophin expression, making any part of the re-expressed molecule potentially immunogenic. Figure generated via BioRender.

In contrast, the mini/micro dystrophin and full-length dystrophin cause immune responses in DMD patients not expressing any dystrophin from the mutant gene because these proteins contain antigenic epitopes that were not present when patient’s immune system developed. Therefore, they may be recognised as nonself and targeted by the patient’s immune system. This seemingly predictable explanation is, in fact, much more layered and complicated in all but rare dystrophin-null patients with large DMD gene rearrangements that eliminate the expression of all dystrophins, where any part of the re-expressed protein can contain immunogenic epitopes (Fig. 1).

Most DMD patients lack full-length dystrophins only because deletions and duplications cluster in hotspot regions encompassing exons 45–55 and 3–9, respectively with ~47% and 7% of patients having these mutations [38]. Therefore, most patients express the C-terminal domains of dystrophins, and these portions of the re-expressed therapeutic product are unlikely to be immunogenic because the patient’s immune system recognises these domains as self.

Patients with out-of-frame mutations in the exons 45–55 hotspot do not produce functional dystrophin but may transiently express truncated N-terminal peptides, which, albeit unstable, may be presented to the immune system, resulting in some tolerance to this region in these patients. In contrast, in patients with exons 3–9 hotspot mutations, such N-terminal peptides are not made, and the antigenic epitopes in the dystrophin N-terminus could be immunogenic. Clinical trial data confirm this scenario, where significant side effects (myositis) occurred in some such patients [37]. Recent analysis narrowed down the T cell-mediated responses to be directed against specific peptides encoded by exons 8 and 9 of the DMD gene [39]. These findings confirm that antigenic epitopes within micro-dystrophins may have high immunogenic potential.

The STOP mutations are evenly distributed across the DMD exons [40]. Patients with such mutations located in the distal part may potentially express traces of dystrophin owing to spontaneous readthrough, as can patients with mutations producing non-functional, rapidly degraded dystrophin proteins. Interestingly, mutant dystrophin transcripts are not eliminated by nonsense-mediated decay, a mechanism normally responsible for the degradation of mutant mRNAs, but rather, an unusual mechanism causes a reduction in transcript levels [41]. The persistence of mutant out-of-frame dystrophin transcripts can increase the likelihood of their misreading, leading to new epitopes.

Moreover, various mini/micro-dystrophins re-expressed through exon skipping or gene therapy have N-spliced-to-C-junctional epitopes (Fig. 1) that are absent in normal dystrophins and can vary from mini/micro dystrophins endogenously expressed in patients [33, 42]. These junctional epitopes are becoming potential neoantigens.

Indeed, even the smallest sequence differences can be detected. The targeting of full-length human dystrophin in the muscle of adult mdx mice, which lack the full-length isoforms, led to only transient expression, which was rapidly extinguished due to responses against dystrophin, which coincided with the development of myositis. However, an anti-dystrophin immune response was also observed following human dystrophin expression in dystrophin-expressing wild-type mice. These findings indicate that even small differences between self and exogenous dystrophin proteins can induce an immune response [32, 43].

The immunogenic potential and anti-dystrophins responses in the DMD population can involve multiple factors. Approximately 8% of patients present preexisting cellular immunity to dystrophin, which is not eliminated by steroid treatment [44]. Spontaneously appearing mini-dystrophins, typically in revertant fibres, can contribute to immunisation [45]. Revertant fibres (RFs) are rare myofibers that express dystrophin and are found in the muscles of patients and animal models of DMD. Their name indicates that these fibres “revert” to a non-dystrophic phenotype. RFs are believed to arise from spontaneous genetic events, such as but not necessarily exclusively exon skipping, which restore an open reading frame and allow the production of internally truncated Becker-type mini/micro-dystrophins [46]. The number of natural RFs increases with the number of regeneration‒degeneration cycles [47], but their presence is never sufficient to halt disease progression, possibly not only because of the rarity of appropriate exon skipping events but may also involve immune responses against myofibres expressing mini/micro-dystrophins recognised as nonantigens. This possibility has not been investigated.

The memory response from such primed immune system would accelerate the rejection of dystrophin re-expressed *via* therapeutic approaches. Conversely, revertant fibres appearing in pre-/perinatal periods, which is possible albeit rare, could potentially trigger perinatal immune tolerance [48].

Therefore, therapeutic re-expression of mini-dystrophin in such a diverse population of patients is likely to result in extremely variable outcomes, with tolerized patients expressing the transgene long term and those immunised responding strongly and suffering from myositis [37]. Indeed, only some of the patients with mutations involving exons encoding the N-terminal antigenic peptides developed myositis [39]. Clearly, patients’ immune status should be established pre-treatment to mitigate anti-dystrophin responses. However, until recently, no stratification was used, probably due to the overall low number of patients available for trials.

In addition, several disease- and tissue-related factors exacerbate the immunogenicity of re-expressed dystrophin. Skeletal muscle is an immunogenic location; therefore, it is used as a tissue of choice in vaccination [49]. Furthermore, DMD is associated with chronic inflammation [50]. Dystrophic myofiber damage and death trigger the release of damage-associated molecular patterns (DAMPs), which attract infiltrating inflammatory cells (sterile inflammation). Muscle regeneration requires such infiltrating immune cells [51, 52]. However, the essential transition from the initial inflammatory to the anti-inflammatory and pro-regenerative immune response is altered in DMD [51, 53] (reviewed in Rosenberg et al., 2015, Petroff et al., 2022 [54, 55]), and dystrophic muscle becomes chronically inflamed. Inflammation exacerbates immunogenicity [52, 56] although corticosteroids used routinely in DMD treatment should somewhat reduce it [57, 58].

Although the levels of dystrophin required to protect human myofiber are not known, 15%–20% of normal dystrophin are needed to rescue mouse muscles [59]. Therefore, the antigenic load is likely to be significant, and this factor is known to further exacerbate the unfavourable environment [60].

Importantly, the follow-up period must be established carefully because the anti-dystrophin immune response can take time to develop [34]. This may be due to several factors, including not only the epitope differences between the endogenous and exogenous dystrophins and the previous exposure to dystrophin present in RFs but also the vector type and mode of administration. Certain vectors may trigger an immune response to the transgene more rapidly because of their inherent immunogenic properties [61]. Systemic delivery might provoke a faster and more widespread immune response than some forms of localised delivery, while liver targeting may be associated with the liver tolerance effect to antigens [62, 63].

Furthermore, genetic variations, HLA alleles, overall immune status and history of infections, vaccinations, or other immune-modulating conditions can influence the potency and timing of the response [64].

## Mechanisms of immune responses against dystrophin

In the absence of neonatal tolerance against dystrophin, any arising epitopes can be recognised by the immune system as non-self, neo-antigens [36] being presented to T lymphocytes by antigen-presenting cells and triggering both T cell-mediated (CD8^+^ cytotoxic T lymphocytes, CTLs; and CD4^+^ T helper cells, Th) and B cell mediated (antibody) immune responses [65].

**Fig. 2:**
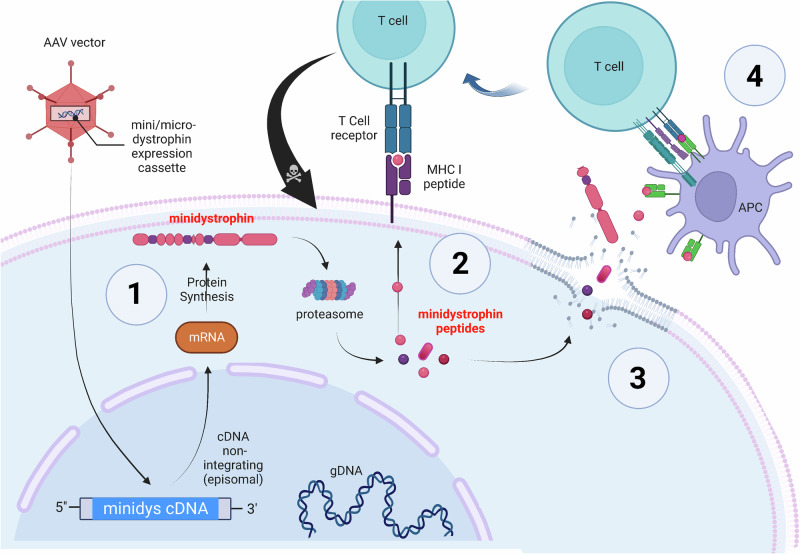
The immune response against re-expressed dystrophin. The targeting vector containing the expression construct transduces dystrophic muscle cells, initiating the expression of mini/micro-dystrophin (1), which can then fulfil its therapeutic role. Like any endogenously expressed protein, a small amount of dystrophin is processed, and MHC class I molecules present peptide fragments derived from dystrophin on the cell surface (2). Note: The assembly process in the endoplasmic reticulum is not shown. When muscle cells are damaged or die, mini-dystrophin, its large fragments, and antigenic peptides are released and taken up by phagocytes and professional antigen-presenting cells (APCs), which subsequently activate a repertoire of T cells that are reactive to dystrophin antigens (4). As a result, all muscle cells expressing dystrophin, even if healthy, are targeted by cytotoxic T cells that recognise the dystrophin peptide/MHC I complex on their surface, leading to the target cell death (indicated by the black arrow). Figure generated with Bio Render.

Dystrophin antigens trigger primarily T-cell-based immune reactions [36, 42, 66]. Accordingly, the MHC class I-dystrophin-peptide complex on the surface of mini-dystrophin-expressing cells becomes the target for T cytotoxic lymphocytes (Fig. 2), which leads to elimination of transgene-expressing cells, cessation of the therapeutic effect [33, 36], and initiating additional tissue damage (e.g., myositis).

Typically, autoimmunity, or the response against own tissues mediated by T cells reactive to “self” antigens, is avoided by the thymic deletion of autoreactive T lymphocytes. The autoreactive T cells which manage to avoid thymic deletion and develop into peripheral naïve T cells undergo anergy of apoptosis upon contact with non-professional antigen-presenting cells and receipt of “signal 1” without the costimulatory ‘signal 2” [refs. 67–69]. The escaping autoreactive T cells, which managed to avoid deletion at these two early steps, become activated and progress to the stage of effector cells. These are controlled by additional factors that include regulatory T(reg) cells, immune checkpoints, such as PD1-PDL1/L2 interaction, or protein-, small-molecule-and metabolic suppressive mechanisms (see below).

In relation to anti-dystrophin immunity, two related mechanisms may be responsible for the induction of responses against one’s own, albeit slightly altered, self-protein.

One of them involves super-agonistic T-cell receptor (TCR) ligands. An example of TCR super-agonist is the mutated MART1 (27L). Compared to the wild-type MART1, it forms a much stronger complex with the MHC class I molecule HLA-A2.001 and the relevant T-cell receptor (TCR). This allows it to activate and expand naïve T cells with low affinity TCR for wild-type MART1, which are neither deleted nor anergized in the thymus, converting them into effector T cells. Once TCR downstream signalling is rewired in activated T cells (enhanced functional avidity), the resulting effector and effector-memory T cells can recognise and kill cells that express nonmutated proteins [70].

In DMD, antigenic peptides derived from dystrophin re-expressed following exon skipping and/or gene therapy may be stronger ligands for the TCR of the non-deleted T cells, which cannot recognise endogenous (mutated) dystrophin peptides in the naïve state. However, once effectively primed by therapeutically-delivered dystrophin and achieving the effector status, these cells can recognise and target any dystrophin.

The second scenario involves a phenomenon known as epitope spreading. It refers to the process by which an immune response initially directed against one protein (protein X) leads to the development of immunity against a different, but associated, protein (protein Y) expressed by the same antigen-presenting cell (APC). This can occur when both proteins are derived from the same dying cell, which is subsequently engulfed by the APC, and the antigens are cross-presented to T cells. Alternatively, epitope spreading can arise even when proteins X and Y are expressed by different cells, provided that the same APC is responsible for the uptake and cross-presentation of both antigens to the immune system [71].

In DMD, such a situation may exist if target dystrophin is expressed in RFs, which also expresses some endogenous mini-dystrophin. Once this myofiber dies (which may take time and explains the delay in response) it is taken up by a local DC (or alternative APC), and it now presents both extraneous and endogenous dystrophin peptides. Since it exchanges bidirectional activating signals with T cells, it becomes activated and develops into mature DC, with high levels of costimulatory molecules and capacity to activate naïve or “anergized” T cells. Once activated and converted to effector cells, these T lymphocytes can now target muscle cells expressing any dystrophin.

Importantly, in addition to these general mechanisms, a DMD-specific mechanism might also play a role. In mdx mice, involution of the thymus alters central immune tolerance [72]. This thymus abnormality not only exacerbates, directly [73] or indirectly, muscular dystrophy symptoms, but T-lymphocytes autoreactive to dystrophin and escaping deletion within dystrophic thymus are likely to boost responses to the re-expressed dystrophin. This abnormality might be behind the unexpectedly high immunogenic potential of the dystrophin protein. Importantly, this involution of the thymus found in dystrophic mice has, thus far, not been confirmed in patients. However, both CD4^+^ and CD8^+^ autoreactive T cells have long been implicated in exacerbating the dystrophic process [74–77]; Reviewed in Coles et al., 2022 [78] and lymphoblast from DMD patients show phenotypic alterations [79].

Regardless of the mechanism involved, the development of immune responses against dystrophin will not only drastically reduce the therapeutic efficacy and potentially exacerbate muscle wasting but also prevent any future treatments that aim to re-express dystrophin with the same or similar epitopes.

## Complementary induction of dystrophin tolerance immunosuppression: Lessons from cancer and chronic infections

The appearance of dystrophin-related new epitopes in dystrophin-naïve immune system of DMD patients bears analogy with chronic viral disease and cancer, where high levels of new antigens evade the elimination by immune system by the complementary action of immunosuppressive and tolerogenic measures [80–84]. Such analogy raises the question of whether the mechanisms promoting viral persistence in chronically infected hosts and caner growth, can be leveraged to enhance the effectiveness of DMD genetic therapy and limit the resulting inflammatory response.

The balance between the activation of effector immunity involving cytotoxic lymphocytes (CTLs), Th1 and natural killer (NK) cells, activated (M1) macrophages and opsonizing antibodies versus suppressive cells, such as regulatory T cells (T_reg_), myeloid-derived suppressive cells and M2 (suppressive) macrophages has been shown to determine the difference between the effectively controlled acute infections and tumour elimination versus the development of chronic viral infection (where virus can persist for months and years), and cancer progression [80–85]. Successful persisting viruses and cancer cells mobilize a wide array of suppressive mediators, and immune checkpoints (PD1/PDL1/PDL2, LAG3, TIM3, PGE2, IDO, IL-10, TGFβ, VEGF, NOS2, arginase), which act as mediators, and often inducers of T_reg_, myeloid-derived suppressor cells (MDSCs) and M2 macrophages [80–84, 86–91], which enable them to reduce the effects of mediators of acute inflammation, such as interferons, IL-12 or IL-18 [refs. 86, 92–97]. Of interest, several of these suppressive factors, in addition to their ability to directly suppress T cell functions, can also promote the developments of T_reg_ and MDSCs known to promote immune tolerance.

Therefore, it is a compelling possibility that, in analogy to the documented ability of multiple viruses and cancers to successfully evade the immune system and maintain long-term expression of foreign proteins in nominally immunocompetent hosts, the same mechanisms can be mobilized in the suppression or prevention of immune responses against dystrophin. Immunosuppression has been shown to prolong transgene expression [98] and therefore could be exploited, especially as it can sometimes trigger antigen-specific tolerance [99]. However, immunosuppression disrupts normal responses against pathogens, increasing the risk for compromised DMD patients. Moreover, as dystrophin has tumour-suppressor properties [18, 19], the risk of immunosuppression promoting malignancy in DMD patients needs to be considered.

## Tolerance induction to dystrophin as an alternative to immunosuppression

Antigen-specific strategies aimed at controlling undesired T-cell and humoral responses to dystrophin and viral vectors through genetic vaccination have shown promise in animal studies [43].

Inducing tolerance to therapeutically expressed dystrophin could significantly enhance the efficacy of treatment while minimising potential risks. Recent advances in the understanding of immune tolerance mechanisms have paved the way for developing strategies that promote long-lasting, antigen-specific tolerance [100]. These innovations hold promise for maximising the therapeutic benefits of dystrophin gene therapy by reducing the likelihood of an adverse immune response and ensuring sustained expression of the therapeutic protein.

Interestingly, known cases of longer-term expression of mini-dystrophin are associated with spontaneously increased numbers of T_reg_ in transduced muscles [101–103]. These findings are relevant and encouraging because an earlier study in mdx mice suggested that it may be difficult to induce tolerance to muscle neoantigens [104].

Alongside T-cell anergy and clonal deletion of antigen-specific T lymphocytes, the upregulation of T_reg_ is a crucial mechanism for establishing immune tolerance [99]. Therefore, leveraging strategies that promote T_reg_ development could be a promising approach to sustaining microdystrophin expression in adult dystrophic muscles, potentially enhancing the long-term efficacy of gene therapies in treating muscular dystrophies.

Indeed, a proof-of-principle study involving co-expression of a strong model antigen (β-gal) with indoleamine 2,3-dioxygenase (IDO1) in *mdx* mouse muscles revealed significantly increased transgene expression, concomitant with significantly elevated T_reg_, reduced inflammatory cells attacking β-gal-expressing muscle and decreased anti-transgene systemic immune responses [105]. This approach exploits the natural mechanism whereby elevated IDO activity converts dendritic cells into tolerogenic antigen-presenting cells that suppress effector T lymphocytes and promote T_reg_ expansion and thereby tolerance (Reviewed in Mellor et al., 2017 [106]). Importantly, IDO1 expression in *mdx* myofibres follows a physiological mechanism in which released IDO1 performs its function [105]. There is no need to directly target immune cells, and the option to express dystrophin and IDO1 from two separate vectors [99] significantly simplifies the therapeutic approach. In the future, bicistronic vectors combining micro-dystrophin and IDO1 could be developed.

Whether IDO represents a promising therapeutic target with clinical relevance needs to be studied, but this immunosuppressive molecule has been implicated in one of the most remarkable examples of tolerance induction, which is maternal tolerance to the semi-allogeneic foetus [107]. Moreover, IDO1 triggers peripheral tolerance [108] induces tolerance to transplanted organs, permits scaling-down immunosuppression [109] and is also involved in neonatal/perinatal tolerance. This latter mechanism results in the immature immune systems of newborns avoiding unnecessary immune responses to maternal antigens; tolerating antigens from the environment, food, and nonharmful microorganisms; and limiting autoimmune responses [110, 111]. Neonatal tolerance is of interest with respect to tolerance induction for gene therapy. Its clear potential [112–114] seems particularly applicable in DMD, where early diagnosis is possible and early treatment would be particularly beneficial. While neonatal tolerogenic protocols could only be considered with a full understanding of the underlying mechanisms, the opportunity is clear. Simple serum creatine kinase screening in newborn boys would allow perinatal diagnosis, and the application of gene therapy in infants capable of developing neonatal tolerance could prevent dystrophic damage. Importantly, even if not curative, such gene therapy might prevent immune responses against subsequent treatments. Tolerance could simultaneously be induced to dystrophin and the selected gene therapy vector, although immunologic ignorance rather than tolerance to viral vectors is also possible [115].

Unfortunately, it should be noted that even when the neonatal tolerance to dystrophin develops, it appears less persistent than tolerance to other, strong antigens [34]. The reason for this is not clear but may involve the aforementioned properties of the protein and the autoreactive T cells escaping deletion within the dystrophic thymus. Taking into account their documented role in promoting tolerance to multiple stronger viral and cancer antigens, the above considerations provide rationale for mobilizing additional suppressive and tolerogenic mechanisms in addition to the IDO pathway, such as PD1/PDL1/PDL2, LAG3, TIM3, PGE2, IDO, IL-10, TGFβ, VEGF, NOS2, arginase, as well as cell therapies involving T_r_eg or MDSCs [80–84, 86–91].

## Conclusions

While restoring muscle dystrophin expression offers significant therapeutic potential, immune responses against the re-expressed protein need to be considered as a challenge to such therapeutic approaches [33, 36, 37, 116]. Hence, evaluation of the immune status of each patient before and during treatment, and the development and testing of new therapeutic approaches to prevent or mitigate anti-dystrophin immunity is worth consideration.
